# Determination of the Influence of Various Factors on the Character of Surface Functionalization of Copper(I) and Copper(II) Oxide Nanosensors with Phenylboronic Acid Derivatives

**DOI:** 10.1021/acs.langmuir.1c02990

**Published:** 2021-12-22

**Authors:** Edyta Proniewicz, Maria Starowicz, Yukihiro Ozaki

**Affiliations:** † Faculty of Foundry Engineering, AGH University of Science and Technology, ul. Reymonta 23, 30-059 Krakow, Poland; ‡ School of Biological and Environmental Sciences, Kwansei Gakuin University, 2-1, Gakuen, Sanda, Hyogo 669-1337, Japan

## Abstract

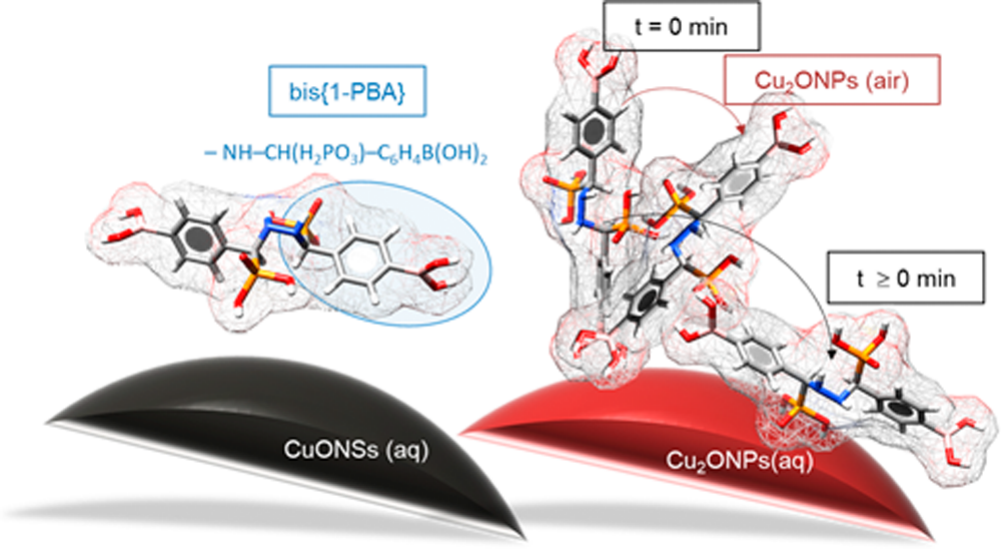

In this work, we attempt to determine the influence of the oxidation state of copper [Cu(I) *vs* Cu(II)], the nature of the interface (solid/aqueous *vs* solid/air), the incubation time, and the structure of N-substituted phenylboronic acids (PBAs) functionalizing the surface of copper oxide nanostructures (NSs) on the mode of adsorption. For this purpose, 4-[(*N*-anilino)(phosphono)-*S*-methyl]phenylboronic acid (1-PBA) and its two analogues (2-PBA and bis{1-PBA}) and the copper oxide NSs were synthesized in a surfactant-/ion-free solution via a synthetic route that allows controlling the size and morphology of NSs. The NSs were characterized by scanning electron microscopy, ultraviolet–visible spectroscopy, Raman spectroscopy, and X-ray diffraction, which confirmed the formation of spherical Cu_2_O nanoparticles (Cu_2_ONPs) with a size of 1.5 μm to 600 nm crystallized in a cubic cuprite structure and leaf-like CuO nanostructures (CuONSs) with dimensions of 80–180 nm in width and 400–700 nm in length and crystallized in a monoclinic structure. PBA analogues were deposited on the surface of the copper oxide NSs, and adsorption was investigated using surface-enhanced Raman spectroscopy (SERS). The changes in the orientation of the molecule relative to the substrate surface caused by the abovementioned factors were described, and the signal enhancement on the copper oxide NSs was determined. This is the first study using vibrational spectroscopy for these compounds.

## 1 Introduction

Arylboronic acids are a class of chemical compounds commonly used in modern synthesis to form C–C and C-heteroatom bonds.^1^ These acids exhibit a reversible coordination profile that is used as a tool for the construction of stimulus-dependent biconjugates used in pharmaceuticals (e.g., antibiotics^2^), polymers,^3^ organic synthesis, electrochemistry, catalysis,^4^ materials chemistry (e.g., to obtain predictably organized crystalline materials^5^), or separation processes.^6,7^ Derivatives of phenylboronic acid (PBA) are used in medicine, for example, in selective drug delivery,^8^ live cell imaging,^9^ cancer treatment (e.g., in the boron neutron capture therapy^10^ and for efficient tumor-targeted chemotherapy with doxorubicin–PBA nanocomplexes^11^ and low-molecular-weight gels based on PBA derivatives^12^), in enzyme and HIV inhibition.^10,13^ PBA are also used in the development of new fluorophores and chemical sensors for glucose in blood^14,15^ or other body fluids.^16^

The use of PBA in the treatment of diabetes is based on their specific binding to 1,2-diols or polyols and the formation of reversible covalent PBA/diol complexes.^17^ The formation of boronic acid esters is favored near or above the p*K*_a_ of boronic acid. In order to modify the p*K*_a_ of PBA and their efficiency in ester formation, many attempts have been made to synthesize various substituted PBA derivatives.^18^ For example, it was found that the addition of electron-withdrawing groups to the aromatic ring can lower the p*K*_a_ by inductive effects, while the addition of electron-donating substituents can increase the p*K*_a_.^18^ Wulff et al. found that the addition of a nitrogen atom can facilitate the formation of boronate esters.^19^ On the other hand, the placement of the carbonyl group facilitates the formation of the boronate ester over almost the entire pH range due to the interaction between boron and carbonyl oxygen.^20−22^ However, compounds containing a carbonyl group have a two-dimensional structure, and the lone pair of electrons of the atoms adjacent to the carbonyl group interacts quite strongly with this group. A similar conjugation is not as pronounced in phosphates with a tetrahedral configuration. Therefore, (amino)phosphonic groups are increasingly used in place of carbonyl groups in the rapidly developing field of biochemistry;^23−25^ although, much work remains to be done in this area. For the abovementioned reasons, we have synthesized N-substituted 4-[(*N*H-R)(phosphono)-*S*-methyl]phenylboronic acids for our research.

The development of metallic nanostructures (NSs), such as Ag, Au, and Zn nanoparticles (NPs) and semiconductor quantum dots surface modified with PBA derivatives also contributed to the exploration of the use of the NSs for the dynamic quantification of glucose in a physiologically important concentration range of 0–20 mM and pH 7.4,^26,27^ for the self-regulatory delivery of insulin at a physiological pH,^28^ and for the detection of sialic acid as a diagnostic and therapeutic agent in cancer.^29−33^ Despite many studies, both the description of the adsorption mode of PBA derivatives and the change of adsorption under the influence of different environmental conditions and the use of copper NPs have been rather neglected, although Cu has a greater biological significance than Ag or Au.^34,35^ Accurate adsorption characteristics of PBA derivatives on the surface of NSs is crucial because changes in the intensity of PBA modes can be misinterpreted. That is, changes in the intensity of PBA signals are interpreted quantitatively (e.g., low-intensity signal—low compound concentration, high-intensity signal—high compound concentration) without taking into account the fact that the intensity changes can be associated with a change in the orientation of PBA on the metal surface. Such errors may lead to decreased medical relevance of surface-modified NPs with PBA.

**Figure 1:**
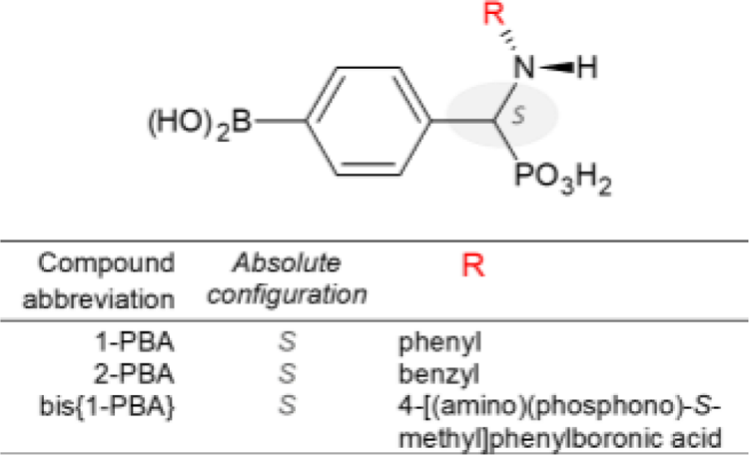
Schematic representation of the chemical structures of the investigated 4-[(*N*H-R)(phosphono)]-*S*-methyl]phenylboronic acids.

The importance of PBA, the enhanced catalytic activity of copper oxides (as Cu is rapidly oxidized under physiological conditions) in the destruction of cancer cells^36−38^ and the advantages of surface-enhanced Raman spectroscopy (SERS)—a technique used in a variety of fields,^39−49^ which allows us to describe the behavior of a selected molecule at the solid/liquid and solid/air interfaces—led us to study the adsorption of N-substituted 4-[(*N*H-R)(phosphono)-*S*-methyl]phenylboronic acid and the changes in adsorption due to changes in the chemical structure of the substituent R– (see Figure 1), oxidation state of Cu (copper(I) (Cu_2_O) *vs* copper(II) (CuO)), and interface type (solid/liquid *vs* solid/air).

## 2 Materials and Methods

### 2.1 Synthesis of N-Substituted 4-[(NH-R)(phosphono)-*S*-methyl]phenylboronic Acids

4-[(*N*-Anilino)(phosphono)-*S*-methyl]phenylboronic acid (1-PBA), 4-[(*N*-benzylamino) (phosphono)-*S*-methyl]phenylboronic acid (2-PBA), and bis{4-[(*N*-anilino)(phosphono)-*S*-methyl]phenylboronic acid} (bis{1-PBA}) (Table 1) were synthesized according to the procedure described previously.^50^ The purity and chemical structure of the compounds were verified by ^1^H, ^13^C, ^31^P, and ^11^B NMR (Bruker Avance DRX 300 MHz spectrometer, Bruker Polska, Poznań) and ESI-MS spectroscopy (Bruker MicrOTOF-Q spectrometer, Bruker Polska, Poznań).

**Table 1:** Wavenumbers (the Position of the Bands Differs Slightly between the Spectra of the Studied Compounds) and Band Assignment for N-Substituted 4-[(*N*H-R)(Phosphono)-*S*-methyl]phenylboronic Acids Adsorbed on the Surfaces of CuONSs and Cu_2_ONPs^72−74^

[cm^–1^]	band assignment	[cm^–1^]	band assignment	[cm^–1^]	band assignment
422	ρ_r_(BO_2_)	881	ν(P–O/C) + ρ_b_(POH) + δ(CPN)	1223	ν(P=O)
525	γ(HOBC) + (HOBO)/ν_16b_	930	ν(P–O),/ν(C–P/C/N),/ν_17b_ [B_1_]	1266	ν(P=O) + ν_3_
551	ν_16b_ [B_1_]/γ(HOBC)/HOBH)/(HOBO)	989	ν_12_	1286	ν(P=O) + ν_4_
584	ρ_s_(NC(H)_2_C) + δ(CC(P)N)	1005	ν_12_	1366	ρ_w_(CH_2_) + ν(B–O)
622	ν_6b_ [B_2_]	1012	δ(BOH)	1386	ν(B–O)
632	δ(CBO_2_) + δ(CC(P)N)	1034	ν_18a_	1444	ν_19b_ + ν(B–O)
665	ρw(CPO)/δ(CC(P)N)	1045	ν_18b_ + ν(C–B)	1475	ρ_r_(CN(H)C)
711	δoop(CBO2) + ν(C–B)	1075	ν(B–OH)	1544	ν_8b_
721	δoop(CC(B)C)_ϕ_	1096	δ(BOH) + ν(B–O)	1580	ν_8b_
760	δ(ring)	1127	ν(C–N) + δ(NH)	1592	ν_8a_
789	ν(C–B) + ν(B–O)/ν_1_	1178	ν_15/9a_	1618	ν_8a_
807	δ(C_α_N(H)C)	1203	ν_7a_		

### 2.2 Synthesis of Colloidal Cu_2_ONPs and CuONSs

Copper(I) oxide (cuprous oxide, Cu_2_ONPs) and copper(II) oxide (cupric oxide, CuONSs) nanostructures (NSs) were prepared by chronoamperometry (at room temperature using a VoltaLab potentiostat PGZ301 and at a constant electrode potential of 0.8 V for 4 h).^51,52^ 0.1 M aqueous solution of lithium chloride (LiCl; from Sigma-Aldrich) was freshly prepared and used for CuONS synthesis, while an ethanolic LiCl solution with 10% water was freshly prepared and used for the synthesis of Cu_2_ONPs. The electrochemical treatment was carried out under an inert atmosphere by slowly bubbling the solution with argon gas in a conventional three-electrode cell with a platinum wire as a counter electrode and an Ag/AgCl (1 M KCl) electrode as a reference electrode (the potential is indicated against this electrode). A copper rod served as the working electrode. Before electrochemical treatment, metallic copper (99.99% Cu) was polished with sandpaper to reduce the grain size and then purified in anhydrous ethanol (99.8%; from Sigma-Aldrich). The precipitated product was in the form of orange Cu_2_ONPs and brown CuONSs.

### 2.3 Ultraviolet–Visible Spectrum Measurements

The ultraviolet–visible spectra (UV–vis) spectra of an aqueous sol and a sample/sol system, measured after 180 min of mixing, were recorded using a LAMBDA 25 UV–vis spectrometer.

### 2.4 Scanning Electron Microscopy Measurements

The scanning electron microscopy (SEM) images of an aqueous sol were acquired using a SEM instrument, model S-5000 (Hitachi Ltd., Japan), operated at 20 kV.

### 2.5 X-ray Diffraction Measurements

X-ray diffraction (XRD) patterns were recorded using a Rigaku UltimaIV X-ray diffractometer (Rigaku Co., Japan) with Cu K_α_ (λ = 1.542 Å) radiation at 40 kV and 40 mA in the range of 20–80° (2θ) with a step of 0.02.

### 2.6 Raman and SER Measurements

Aqueous solutions of the studied compounds were prepared by dissolving each compound in deionized water (18 MΩ·cm^–1^; sample concentration 10^–4^ M). 10 μL of the sample solution was mixed with 20 μL of aqueous sol solution. The 20 μL of the sample/sol mixture was applied to a glass plate, and the SERS spectra were recorded (no measurements were made for the dried droplet). The spectra were recorded three times at three different locations on each surface.

The Raman and SERS spectra were recorded using a HoloSpec f/1.8i spectrograph (Kaiser Optical Systems Inc.) equipped with a liquid-nitrogen-cooled CCD detector (Princeton Instruments). The 785.0 nm line of a NIR diode laser (Invictus) was used as the excitation source. The laser power at the sample position was set to ∼15 mW. The typical exposure time for each SERS measurement was 40 s with four accumulations. The spectral resolution was set to 4 cm^–1^. The SERS spectra of a given adsorbate on a given substrate were almost identical, except for small differences (up to 5%) in some band intensities. No spectral changes that could be associated with the decomposition of the sample were observed in these measurements.

### 2.7 Spectral Analysis

Spectral analysis was performed using a GRAMS/AI program (Galactic Industries Co., Salem, NH).

Several unseparated bands were fitted using the GRAMS/AI program (Galactic Industries Co., Salem, NH). A 50/50% Lorentzian/Gaussian band shape was assumed and fixed for all bands.

## 3 Results and Discussion

### 3.1 Properties of Cu_2_ONPs

**Figure 2:**
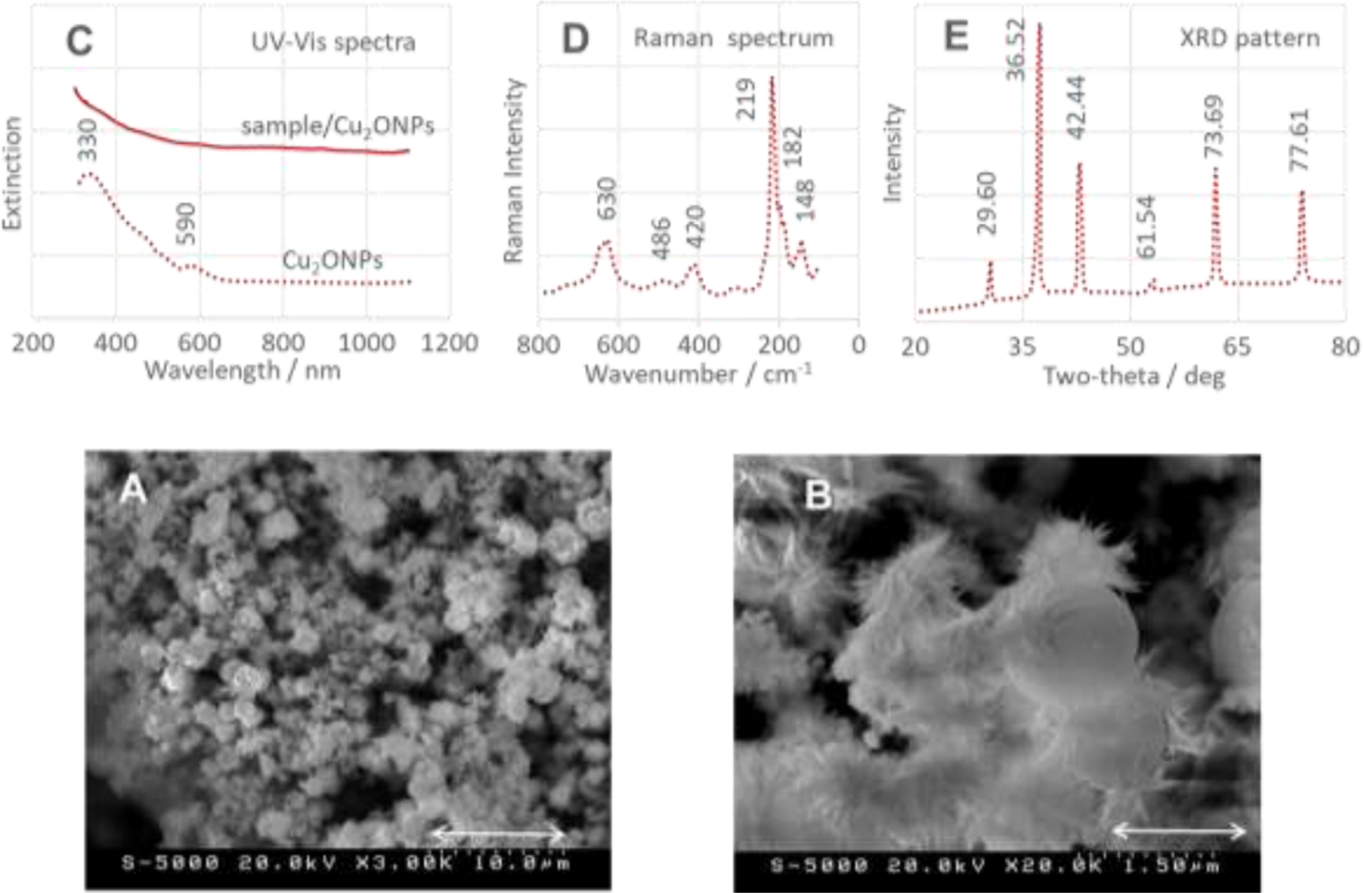
(A,B) SEM images of Cu_2_ONPs (measurement conditions: (A) 20.0 kV, ×3.00 K, scale 10.0 μm; and (B) 20.0 kV, ×20.0 K, scale 1.5 μm), (C) excitation spectra (UV–vis) of aqueous solution of Cu_2_ONPs (dashed red trace) and a sample/Cu_2_ONPs mixture (solid red trace), (D) Raman spectrum of Cu_2_ONPs, and (E) XRD pattern of Cu_2_ONPs.

SEM analysis of the bare copper(I) oxide NPs (Cu_2_ONPs) in Figure 2 (A–scale bar 10 μm and B–scale bar 1.5 μm) shows that the NPs have a spherical shape with a size of 1.5 μm to 600 nm. The UV–vis spectrum of Cu_2_ONPs confirms these observations, as two small plasma resonances are observed at 330 and 590 nm (Figure 2C, red dashed line). The first maximum belongs to the band-to-band transition in nanocrystalline Cu_2_O [O^2–^:Cu^1+^ charge-transfer band (O 2p  →  Cu 3d)],^53−55^ while the second absorption is due to the band gap transition of the CuO layer at the surface of Cu_2_O nanocrystals.^53^

The 2θ values and [(*hkl*)] planes in Figure 2E are 29.60 [(110)], 36.52 [(111)], 42.44 [(200)], 61.54 [(211)], 73.69 [(200)], and 77.61 [(311)] (*Pn*3̅*m*; JCPDS no. 78-2076), indicating the formation of a crystallographically pure, standard cubic cuprite structure.^55^

Figure 2D shows a Raman spectrum of Cu_2_ONPs. Characteristic Raman bands of Cu_2_O are observed at 148 (T_1u_ symmetry), 182, 219 (strongest E_u_), 420, 486, and 630 cm^–1^ (T_1u_) and are in agreement with data from literature.^56−58^ The 148 and 219 cm^–1^ spectral features are due to rotations of the Cu tetrahedron around its center. The 630 cm^–1^ band is attributed to an out-of-plane vibration of the Cu and O sub-lattice and, like the 148 cm^–1^ band, is activated by defects.

### 3.2 Properties of CuONSs

**Figure 3:**
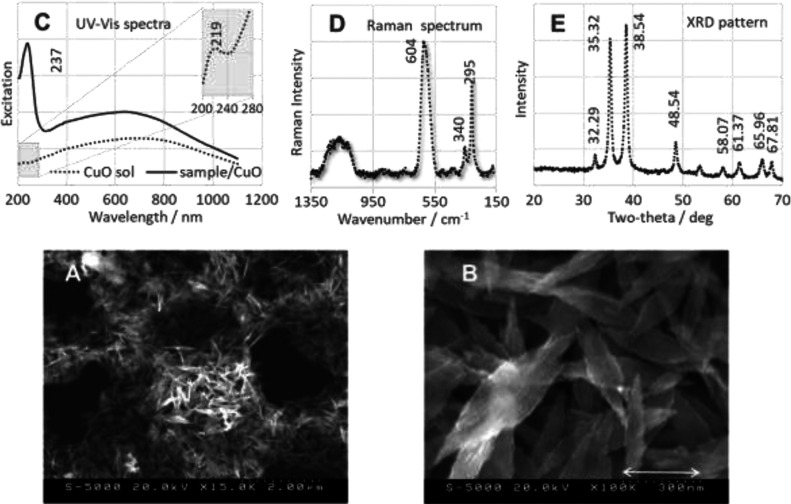
(A,B) SEM images of CuONSs (measurement conditions: (A) 20.0 kV, ×15.0 K, scale 2.0 μm; and (B) 20.0 kV, ×100.0 K, scale 300 nm), (C) excitation spectra (UV–vis) of aqueous solution of CuONSs (dashed black trace) and a CuONSs/sample mixture (solid black trace) used in this work, (D) Raman spectrum of CuONSs, and (E) XRD pattern of CuONSs.

SEM images of CuONSs obtained by the anodic dissolution of Cu are shown in Figure 3 at different magnifications (A— scale bar 2 μm and B—scale bar 300 nm). These images show that the monodisperse CuONSs have a leaf-like structure with average dimensions of 80–180 nm in width and 400–750 nm in length. Moreover, image B shows that these structures are composed of small spherical particles that are self-aligned, which is consistent with previous data on the directional growth of CuO nanocrystals along the axis.^59−61^ Monodisperse CuONSs form a honeycomb-like skeleton consisting of interconnected networks of sub-micrometer pores 2–3 μm in diameter and 1–1.5 μm thick (Figure 3A).

A UV–vis spectrum of the bare leaf-like CuONSs (black dashed line) and that of the sample adsorbed on their surface (sample/CuONSs; black solid line) are also shown in Figure 3. No optical absorption band at ∼290 nm is seen in the UV–vis spectrum of the bare, large-area, self-assembled CuONSs.^6,62^ However, a weak absorption at 219 nm is observed in this spectrum (Figure 3C, black dashed line), which is attributed to the direct transfer of electrons.^63−66^ The spectrum of the sample/CuONSs (Figure 3C, black solid line) shows a broad plasmon resonance with a maximum at about 237 nm. This band is probably due to the π–π* electronic transition of the aromatic C=C groups of the molecule and/or the electrostatic interaction between the CuONS surface and the molecule deposited on this surface.^67,68^

The Raman spectrum of CuONSs in Figure 3D shows the formation of the pure monoclinic CuO structure (space group *C*2/*c*).^59,69^ For the monoclinic structure with two CuO molecules in the unit cell, the group theory predicts six IR-active (of 3A_u_ + 3B_u_ symmetry) and three Raman-active (A_g_ + 2B_g_; of oxygen vibrations) optical modes.^70^ The three Raman-active modes are observed at 295 (A_g_), 340 (B_g_), and 604 cm^–1^ (B_g_) (Figure 3D).

The XRD pattern used to characterize the size and size distribution of the crystalline CuO domains is shown in Figure 3E. The diffraction peaks at 2θ = 32.29, 35.32, 38.54, 48.54, 53.40, 58.07, 61.37, 65.96, and 67.81° are indexed as [110], [1̅11]/[002], [111]/[200], [202̅], [020], [202], [113̅], [311̅], and [220] planes of the pure CuO nanophase with a monoclinic structure (JCPDS no. 48-1548).^71^ The pronounced intensity of the diffraction peaks indicates the highly crystalline nature of the CuONSs.

### 3.3 Influence of the Oxidation State of Copper on Surface Functionalization: CuONSs *versus* Cu_2_ONPs

**Figure 4:**
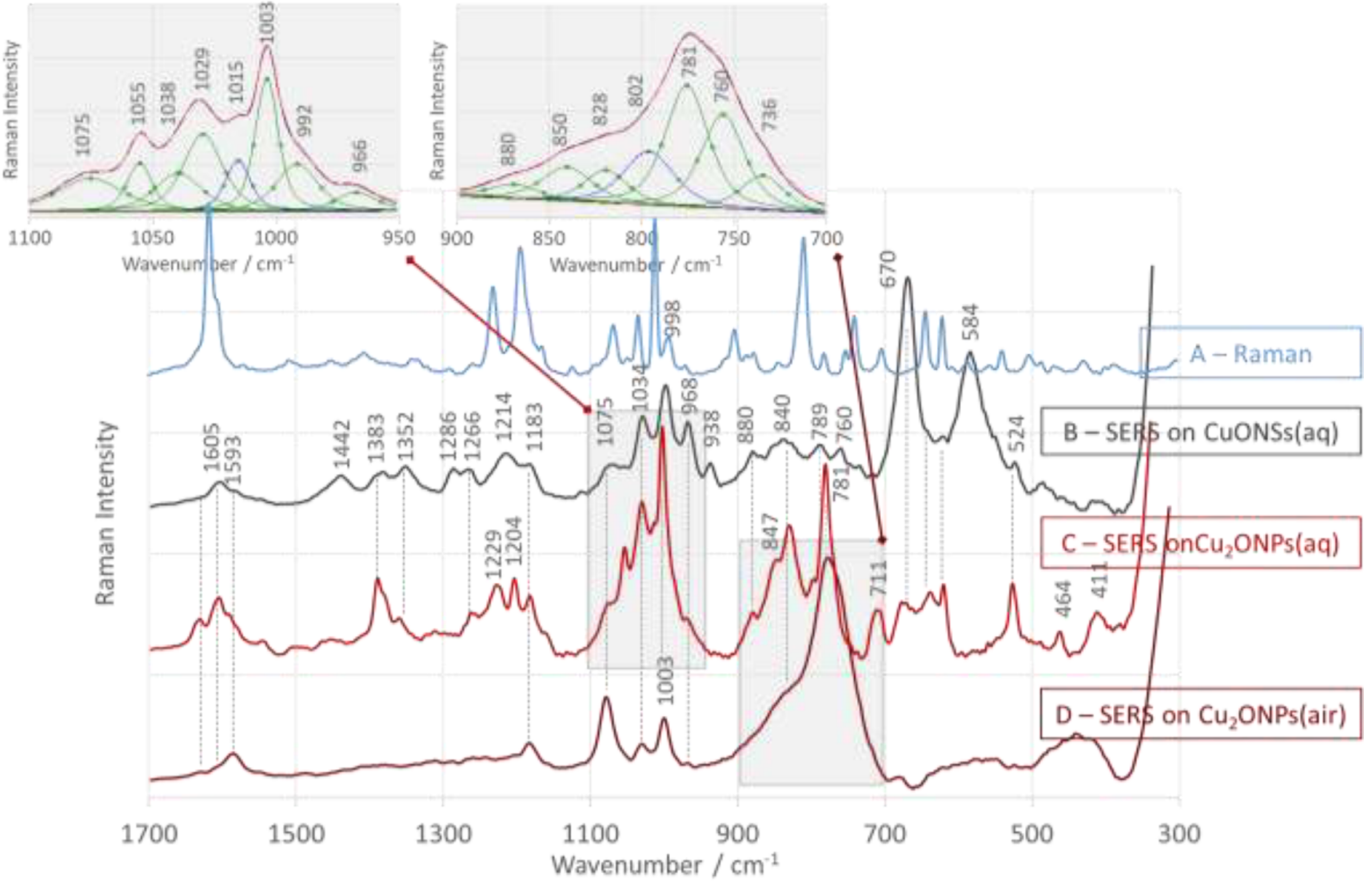
Raman (A) and SERS spectra of 4-[(*N*-anilino)(phosphono)-*S*-methyl]phenylboronic acid (1-PBA) adsorbed at water/CuONSs (B), water/Cu_2_ONPs (C), and air/Cu_2_ONPs (D) interfaces in the spectral range of 1700–300 cm^–1^.

**Figure 5:**
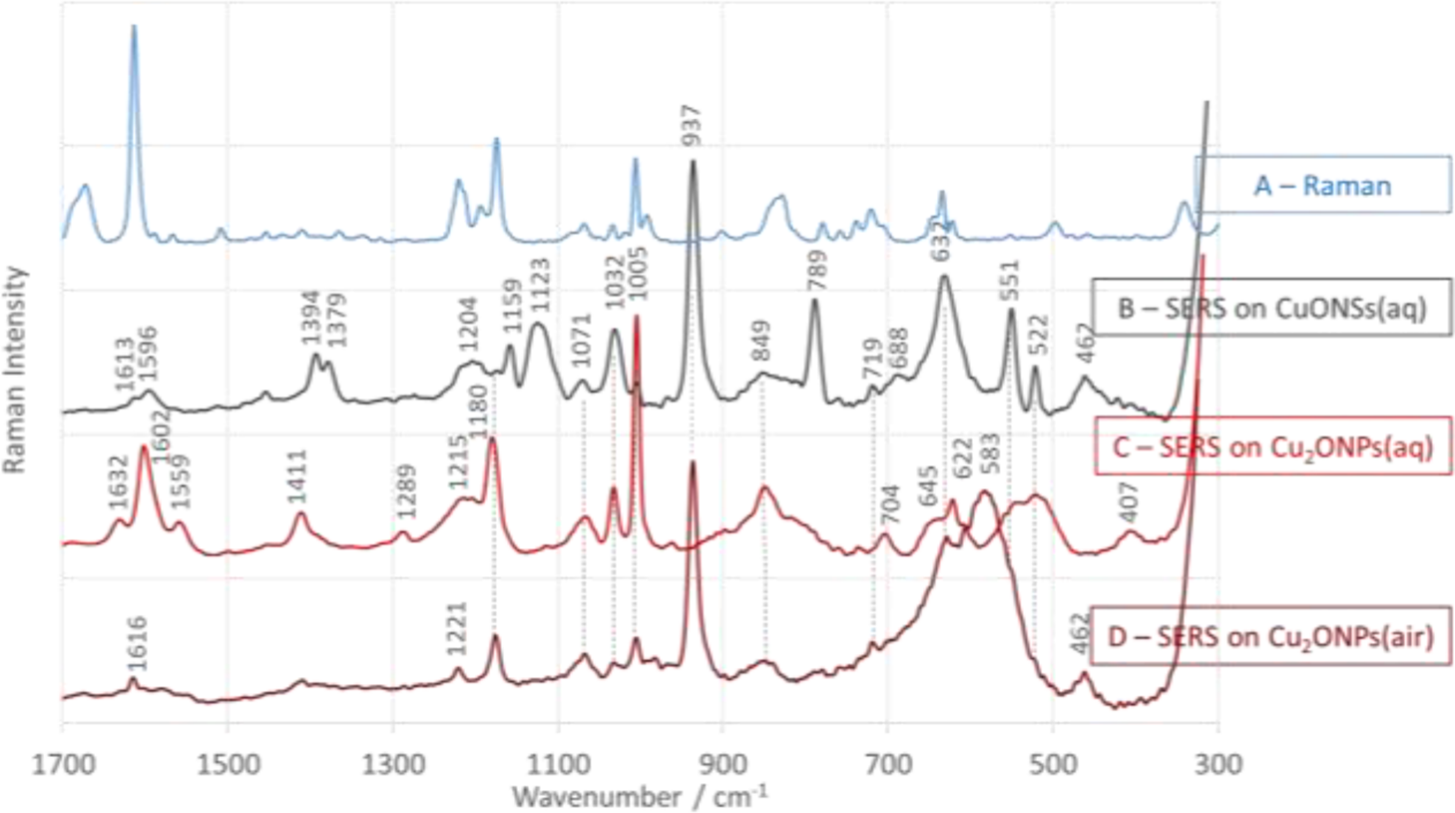
Raman (A) and SERS spectra of 4-[(*N*-aminobenzylo)(phosphono)-*S*-methyl]phenylboronic acid (2-PBA) adsorbed at water/CuONSs (B), water/Cu_2_ONPs (C), and air/Cu_2_ONPs (D) interfaces in the spectral range of 1700–300 cm^–1^.

**Figure 6:**
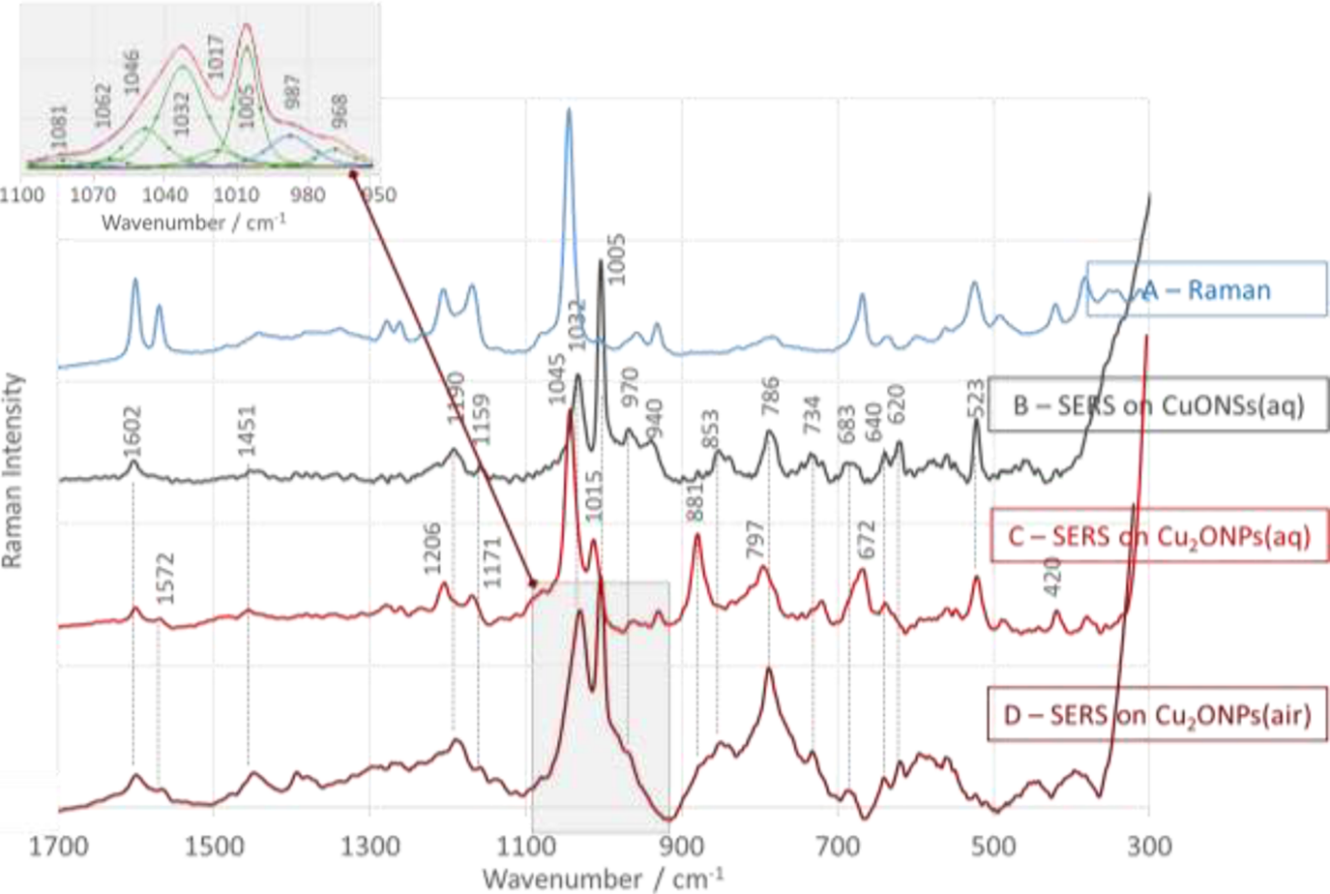
SERS spectra of bis{4-[(*N*-anilino)(phosphono)-*S*-methyl]phenylboronic acid} (bis{1-PBA}) adsorbed at water/CuONS (A), water/Cu_2_ONP (B), and air/Cu_2_ONP (C) interfaces in the spectral range of 1700–300 cm^–1^.

The Raman (Figures 4A–6A, blue traces) and SERS spectra at an excitation wavelength 785.0 nm of 1-PBA, 2-PBA, and bis{1-PBA} adsorbed at the surface of CuONSs (Figures 4B–6B, black traces) and Cu_2_ONPs (Figures 4C–6C, red traces) in aqueous solution at pH = 7 are shown in Figures 4–6. The assignment of bands in these spectra (mainly based on the density functional calculation for PBA derivatives^72^) is given in Table 1.

As can be seen in Figure 4B, the SERS spectrum of 1-PBA adsorbed from aqueous solution on the surface of CuONSs is dominated by two broad bands at 584 (full width at half maximum, fwhm = 58 cm^–1^) and 670 cm^–1^ (fwhm = 33 cm^–1^) (see Table 1 for the assignment of these bands). These bands are not visible in the corresponding Raman spectrum (Figure 4A). Therefore, it can be assumed that the 1-PBA molecule binds to the CuONSs surface via the fragment −C(N)PO–. This implies that the free electron pair on the oxygen (of the phosphonic acid group) and the nitrogen atoms are in direct contact with the substrate surface. Considering the sp^2^ and sp^3^ hybridization of boron and oxygen and nitrogen, respectively, it is expected that the phenyl ring (Ph) adopts an inclined orientation with respect to the surface of the substrate. This arrangement confirms a shift of −5 cm^–1^ in the wavenumber and a broadening of 3 cm^–1^ in the bandwidth of the SERS signal at 998 cm^–1^ (compared to the values in the corresponding Raman spectrum (Figure 4A)). This statement can be supported by the enhancement of the other bands due to the aromatic ring modes (at 1034, 1183, 1214, 1587, and 1607 cm^–1^). However, there is no evidence of interaction of the boronophenyl ring (Ph_B(OH)2_) with the CuONS surface (weak or no bands due to the vibrations of the boronic acid group).

Together with the change in the oxidation state of copper (CuO *vs* Cu_2_O), a change in the character of the 1-PBA interaction with the substrate surface is observed. For 1-PBA adsorbed on Cu_2_ONPs (Figure 4C), the enhancement of two groups of ν_12_, ν_18a_, ν_8b_, and ν_8a_ modes is observed (at 1003^c^, 1038^c^, 1593, and 1611 cm^–1^ and at 992^c^, 1029^c^, 1582, and 1605 cm^–1^; where c represents the curve-fitted bands), indicating the contact of two aromatic rings with the Cu_2_ONP surface. The strong intensity of the SERS signal at 1003 cm^–1^ combined with the absence of a shift in its wavenumber and a broadening of its width indicates the perpendicular arrangement of a ring with respect to the Cu_2_ONP surface. On the other hand, the weak intensity, downward shift in the wavenumber (Δ_ν12_ = −8 cm^–1^), and broadening of the width (Δ_fwhm_ = 4 cm^–1^) of the spectral feature at 992^c^ cm^–1^ indicate a nearly horizontal orientation of the second ring.

With the change of the substrate from CuONSs to Cu_2_ONPs, the bands originating from the vibrations of the P=O fragment lose intensity or disappear (at 1286 and 670 cm^–1^) (Figure 4C). The disappearance of the band at 584 cm^–1^ (on CuONSs) and the increase of intensity at 524, ∼600, and 1383 cm^–1^ in Figure 4C (on Cu_2_ONPs) are also observed. On the other hand, the wavenumber of the SERS signal at 789 cm^–1^ [ν(B–C) + ν(B–O)] shifts to 781 cm^–1^, and the intensity increases. To explain these observations, it can be assumed that the phosphonic acid fragment is away from the Cu_2_ONP surface and the sp^3^ orbital of the boron oxygen atom occupied by the free electron pair has a vertical orientation with respect to this surface. In this orientation, the Ph_B(OH)_2__ ring is tilted by about 70° with respect to the surface normal, that is, it adopts a nearly horizontal orientation on the surface of the substrate, while the Ph ring adopts a nearly vertical orientation.

In the case of 2-PBA adsorbed on CuONSs and Cu_2_ONPs, the change in the oxidation state of copper also leads to changes in the adsorption mode. For this molecule immobilized on Cu_2_ONPs (Figure 5C), a strong enhancement of the bands attributed to the vibrations of the aromatic ring (in particular, the 1005 cm^–1^ band), together with the absence of the wavenumber shift and band broadening compared to the SERS spectrum on CuONSs, is evidence of the vertical arrangement of the aromatic ring on the Cu_2_ONP surface. Also, the absence of spectral features at 937, 789, and 551 cm^–1^ for 2-PBA on Cu_2_ONPs (Figure 5C), which are the most intense bands in the SERS spectrum of this molecule on CuONSs (Figure 5A), indicates the absence of C–N···Cu_2_ONP, P–O···Cu_2_ONP, and B(OH)__2__···Cu_2_ONP interactions.

The SERS spectrum recorded immediately after the addition of bis{1-PBA} to the CuONSs sol (Figure 6B) is dominated by the Ph_B(OH)_2__ ring modes (ν_18a,_ ν_19b_, ν_9a_, ν_18a_, ν_12_, ν_1,_ ν_6b_, and ν_16b_ (see Table 1)),^73^ of which ν_12_ is the most intense. Again, the changes in the intensity (Raman *vs* SERS) of these bands indicate the presence of the ring in the perpendicular orientation on the CuONS surface. Moreover, the absence of the characteristic δ(BOH) mode (at about 1075 cm^–1^) indicates that the −B(OH)_2_ group is not involved in the interaction with the substrate, so that the 786 and 523 cm^–1^ bands are due to ring vibrations. When the substrate is changed from CuONSs (Figure 6B) to Cu_2_ONPs (Figure 6C), there is a strong enhancement of the band at 1045 cm^–1^ accompanied by SERS signals of the intermediate intensity at 1015, 881, 797, and 672 cm^–1^ (see Table 1 for band assignments). These bands indicate the adsorption of bis{1-PBA} by the Ph_B(OH)_2__ ring, which is arranged more or less horizontally with respect to the Cu_2_ONP surface, allowing the observation of a strongly enhanced band at 1045 cm^–1^ (containing a contribution from the C–B vibration) and a weakly enhanced spectral feature at 1005 cm^–1^. In addition, the −C(N)PO···Cu_2_ONP interactions are possible with this ring arrangement.

### 3.4 Effect of Incubation Time on Surface Functionalization

**Figure 7:**
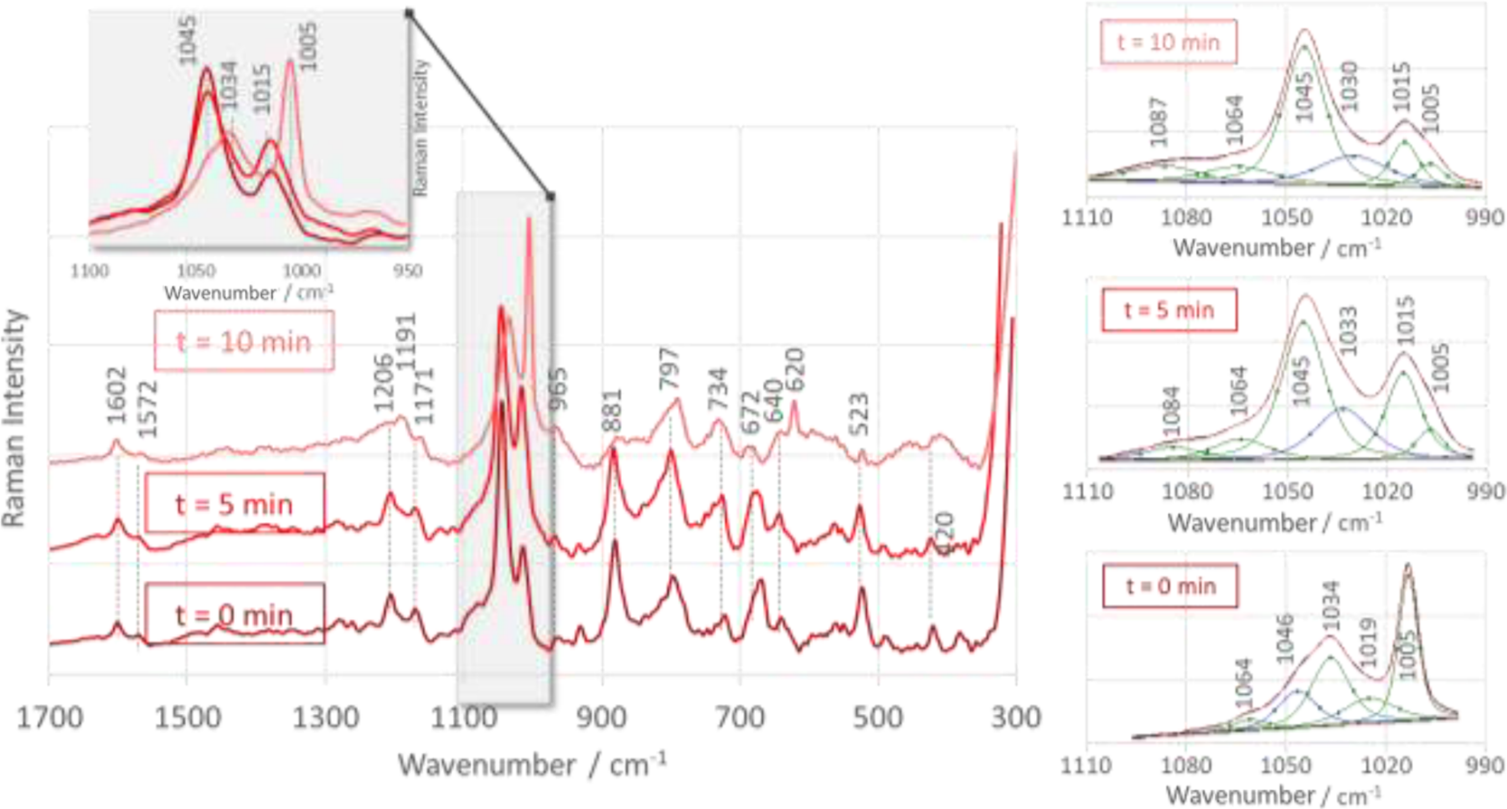
Time-dependent SERS spectra of bis{4-[(*N*-anilino)(phosphono)-*S*-methyl]phenylboronic acid} (bis{1-PBA}) adsorbed at the water/Cu_2_ONP interface in the spectral range of 1700–300 cm^–1^.

The spectra of bis{1-PBA} adsorbed at the Cu_2_ONPs/water interface show spectral changes as a function of incubation time (Figure 7). In the spectrum measured immediately after the adsorption of the compound on the surface of the substrate (*t* = 0 min), the band at 1045^c^ cm^–1^ is the most intense, and the band at 1015^c^ cm^–1^ has an intensity of 30% of the band at 1045^c^ cm^–1^. When the incubation time is *t* = 5 min, the SERS signal at 1045^c^ cm^–1^ decreases, while the band at 1015^c^ cm^–1^ increases in intensity (*I*_1015_/*I*_1045_ = 0.6). Further extension of the incubation time to 10 min leads to a further increase in both the *I*_1015_/*I*_1045_ intensity ratio to 0.7 and the intensity of the spectral feature at 1005^c^ cm^–1^, which becomes the strongest band in the spectrum (4 times stronger than the 1045 cm^–1^ band). Another important change over time in the SERS spectra of bis{1-PBA} adsorbed at the Cu_2_ONP/water interface is the decrease in the intensity of the 881, 797, 672, and 523 cm^–1^ bands. Extending the incubation time beyond 10 min has no effect on the SERS spectrum profile, suggesting that only in the first 10 min of adsorption there is a reorientation of the molecule at the Cu_2_ONP/water interface. This reorientation is the adsorption of an upright position of the molecule, where only one of the Ph_B(OH)_2__ rings is in direct contact with Cu_2_ONPs, versus the flat position, where the Ph_B(OH)_2__ rings are arranged horizontally on the substrate surface and the P–O fragment interacts with Cu_2_ONPs.

### 3.5 Effect of Changes in the Chemical Structure on Surface Functionalization

The 2-PBA differs from the 1-PBA by the −CH_2_– group (Figure 1). In 1-PBA, the amino group is substituted by a phenyl group, whereas in 2-PBA it is substituted by a benzyl group. Along with this structural change, a change in the adsorption mode is observed. In contrast to 1-PBA on CuONSs (Figure 4B), in the SERS spectrum of 2-PBA on CuONSs (Figure 5B): (1) the 1005 cm^–1^ band shows very low intensity, and 1032, 632, and 551 cm^–1^ spectral features are the most intense bands of the phenyl ring, (2) the 937 cm^–1^ SERS signal [ν(C–C/N) + ν(P–O)] is the strongest band of the spectrum, (3) the 670 and 584 cm^–1^ bands disappear, and (4) the 789 cm^–1^ SERS signal is enhanced and has an intensity comparable to that of the 632 cm^–1^ SERS signal. It can be concluded that after the substitution of the phenyl group (1-PBA) by the benzyl group (2-PBA), the Ph ring is either flat on the surface of the CuONSs or is not in direct contact with this surface. To determine which of these two situations is most likely and which of the two aromatic rings interacts with the CuONSs, one must consider the absence of changes in the wavenumber and bandwidth of the 1005 cm^–1^ band and the presence of bands associated with the vibrations of the −B(OH)_2_ fragment (at 789, 632, and 551 cm^–1^). The fact that the SERS signal at 1005 cm^–1^ has a low intensity, with no broadening in width and no shift in frequency, indicates a vertical orientation of the ring at some distance from the substrate surface. On the other hand, the enhancement of the bands of the boronic acid vibrations indicates that the ring interacting with the CuONSs is Ph_B(OH)_2__.

For the further modification of the molecular structure by doubling the fragment of 4-[(*N*-anilino)(phosphono)-*S*-methyl]phenylboronic acid (bis{1-PBA}), a closer contact between the molecule and the CuONS surface can be proposed, maintaining the orientation of the Ph_B(OH)_2__ ring and limiting the contact between the −C(N)PO– fragment and the CuONSs. This conclusion is based on the intense Ph_B(OH)2_ modes (see the Results and Discussion section above) and a slight broadening of the bandwidth for these modes.

In the SERS spectra of the studied analogues adsorbed on the Cu_2_ONP surface, the following changes were observed under the influence of the structural modifications. The two aromatic rings of 1-PBA interact with the substrate surface, which is accompanied by P=O, B–C, and B–O···Cu_2_ONP interactions. The extension of the chain with the −CH_2_– group (in the case of 2-PBA) moves the Ph_(BOH)2_ ring away from the surface and reduces other types of interactions. Substitution of the Ph ring by a fragment of 4-[(*N*-anilino)(phosphono)-*S*-methyl] phenylboronic acid (the bis{1-PBA} case) forces the molecule to bind to the substrate surface with strong P–O···Cu_2_ONPs interactions.

### 3.6 Influence of Interface Type on Surface Functionalization

The change of the interface from Cu_2_ONPs/water (Figures 4C, 5C, and 6C) to Cu_2_ONPs/air (Figures 4D, 5D, and 6D) leads to a change of the spectral profile, that is, the adsorbed geometry. Briefly, for 1-PBA at the Cu_2_ONP/water interface, the very strong 781 cm^–1^ SERS signal is significantly broadened (Δ_fwhm_ = 50 cm^–1^) and has an asymmetric shape. The decomposition of this band shows that it contains two principal components at 781 and 760 cm^–1^ [δ(ring)]. The very intense spectral features at 1003 and 1038 cm^–1^ decrease significantly in intensity compared to those for 1-PBA at the Cu_2_ONP/water interface, while the SERS signals at 992^c^ and 1029^c^ cm^–1^ disappear. This indicates that only one of the rings is in contact with the Cu_2_ONP/air interface in a tilted orientation. Because the bands at 781 and 1075 cm^–1^ are enhanced in the spectrum, it can be assumed that this ring is a Ph_B(OH)_2__ ring.

2-PBA is adsorbed at the Cu_2_ONP/water interface by the vertical Ph ring (see the Results and Discussion section above) (Figure 5C), whereas the ring at the Cu_2_ONP/air interface is not in contact with the substrate surface, as indicated by a slight enhancement of the 1005 cm^–1^ band, which does not shift in the wavenumber and increases in the bandwidth. The fragment −C(N)PO– is responsible for the adsorption of 2-PBA at the Cu_2_ONP/air interface, as confirmed by the pronounced 937 cm^–1^ SERS signal and the broad band with maxima at 632, 622, and 583 cm^–1^ (see Table 1 for band assignments).

In the case of bis{1-PBA} at the Cu_2_ONP/air interface, the intensity of a 1045 cm^–1^ band decreases and is therefore masked by a 1032 cm^–1^ band whose intensity increases as does the intensity of the SERS signal at 1005 cm^–1^. At the same time, the SERS signals at 881 and 797 cm^–1^ are attenuated and enhanced, respectively, and the spectral feature at 523 cm^–1^ disappears. Considering the assignment of the above bands to the modes proposed in Table 1, we can conclude that the change of the interface brings the Ph_B(OH)_2__ ring closer to the surface, with simultaneous positioning perpendicular to the Cu_2_ONP/air interface. The average intensity of the 797 cm^–1^ band also suggests that the B–O has an angular orientation with respect to this interface. For this to be possible, the Ph_B(OH)_2__ ring must be in contact with the substrate via the C_2_–C_3_ atoms of the ring.

### 3.7 Mechanism of Enhancement

The enhancement factor (EF) quantitatively evaluates the effectiveness of the SERS substrate. The most commonly used definition of EF is EF = (*I*_SERS_/*c*_SERS_)/(*I*_RS_/*c*_RS_), where *I*_SERS_ and *I*_RS_ are the Raman intensities of SERS and non-SERS substrates, respectively, while *c*_SERS_ and *c*_RS_ are the analyte concentrations used for SERS and non-SERS substrates, respectively.^75^ For the same analyte concentrations, EF is equal *I*_SERS_/*I*_RS_. The calculated EF is up to 10^6^ orders of magnitude for Ag and Au@SiO_2_, 10^5^ orders of magnitude for Au, 10^4^ orders of magnitude for Cu and Ti; 10^3^ orders of magnitude for ZnO, CuO, Cu_2_O, TiO_2_, and γ-Fe_2_O_3_; and 10^2^ orders of magnitude for Zn and Fe.^76,77^

The mechanism of the enhancement can be predicted from the SERS spectra. When the adsorbate is physisorbed on the metal surface [electromagnetic (EM) mechanism], its SERS spectrum resembles the Raman spectrum of the free molecule.^78,79^ When the adsorbate is chemisorbed on the metal surface (charge transfer (CT) mechanism), the formed adsorbate–molecule complex leads to drastic changes in the wavenumbers and intensities of the SERS bands of the adsorbate compared to the corresponding Raman bands.^78^ The results of Otero and colleagues have shown that the CT mechanism is responsible for the enormous SERS intensity of the ν_8a_ mode, which can be used as a marker band to detect and estimate the enhancement produced by the CT mechanism for an adsorbate with an aromatic ring (e.g., benzene, pyridine, pyridazine, and derivatives).^80−82^ Considering the above information and the fact that (1) the intensity, width, and wavenumber of the adsorbate bands are only slightly changed compared to these values in the Raman spectrum and (2) there is no particular enhancement of the band due to the ν_8a_ mode, it can be concluded that on the tested substrates the EM mechanism is responsible for the signal enhancement.

## 4 Conclusions

Compared to their precursor, boronic acid, PBA derivatives show a stronger and more selective antimigratory response to cancer cells in the short term while decreasing the long-term viability of these cells.^83,84^ These properties make PBA analogues promising compounds for new cancer therapies. This motivates us to search for and develop new PBA analogues that can selectively inhibit the metastatic properties of various cancer cells.

On the other hand, it has been shown that the unique property of PBA analogues is that they can reversibly bind diols in a covalent manner, which allows, for example, the measurement of glucose fluctuations or the recognition of sialic acid (the expression of sialoglycans in neoplastic cells is observed^85^), giving them great potential for therapeutic diagnostics.^86−90^ Non-enzymatic glucose sensors based on copper or copper oxide/hydroxide NPs have also been developed.^91−97^ The presence of copper has been shown to increase the rate of the glucose oxidation reaction and the stability of the sensor itself. It has also been shown that boronic acid groups immobilized on the surface of copper oxide NPs can form reversible covalent bonds with diol groups of glycoproteins on the surface of the microbial cell, which greatly increases the antimicrobial or antifungal activity of these NPs.^98,99^ However, it should be kept in mind that copper oxide NPs need to be strictly regulated due to the toxic effect of Cu(II) ions released into the body, which can cause neurodegenerative diseases.^100^ Therefore, the prepared sensors containing boric acid can be used to detect copper ions.^101,102^

Therefore, not only are PBA analogues being sought after but techniques are also being developed to enable the detection of these molecules, such as the highly sensitive and selective SERS technique. However, the vast majority of these studies focus mainly on the detection capabilities of SERS and are concerned only with the structure of boronic acid derivatives and the nature of the molecular recognition process, ignoring the immobilization of PBA molecules on metal surfaces. Insights into the behavior of immobilized molecules and the intermolecular interactions between the functional groups during molecular recognition are therefore highly desirable for the proper design of SERS sensors. In the absence of such detailed studies, we have performed and described them in this work for a newly developed potential biosensor combining the properties of copper oxide NPs and PBA.

**Figure 8:**
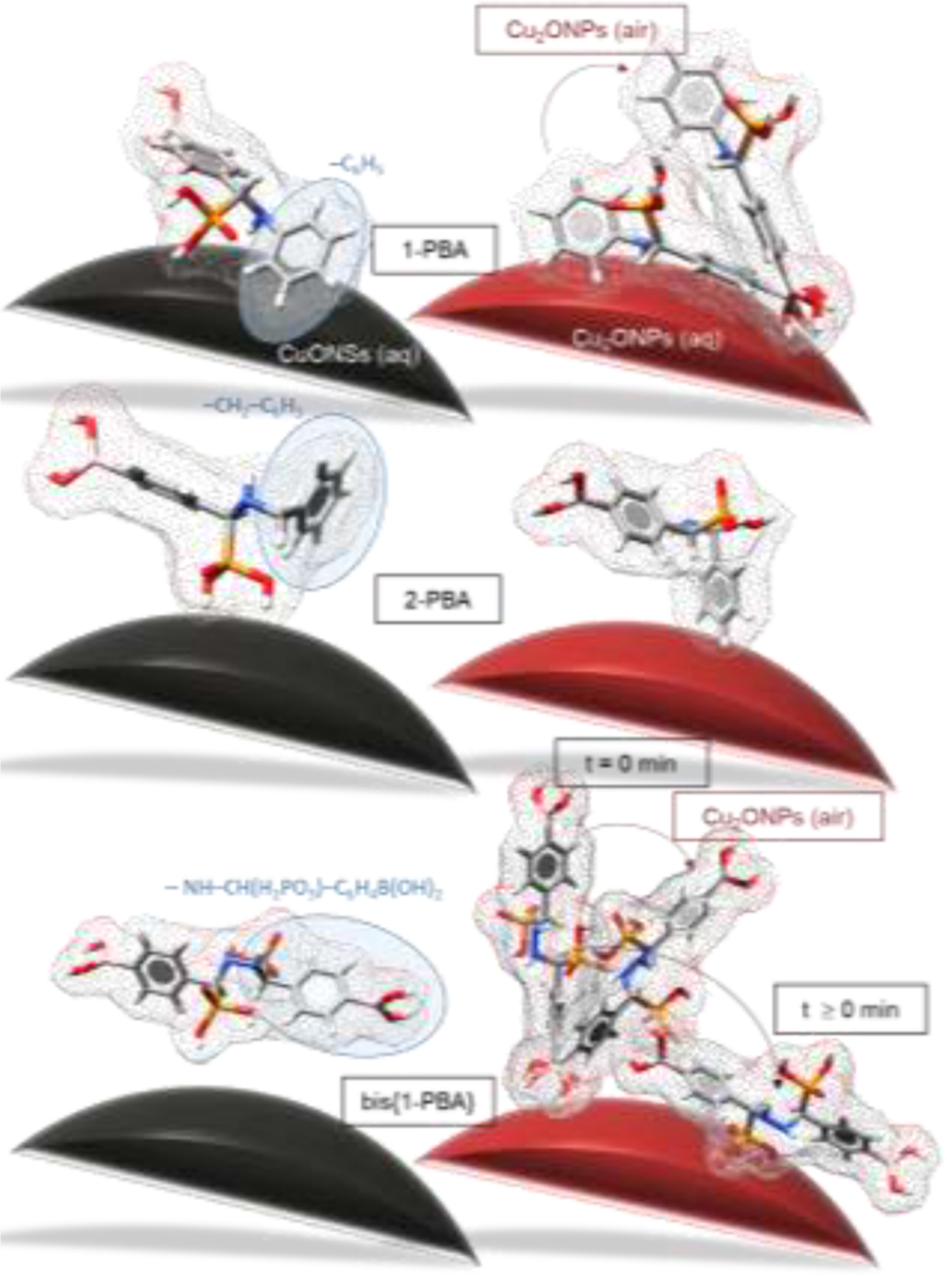
Proposed changes in the adsorption mode as influenced by the oxidation state of copper (Cu(I) *vs* Cu(II)), type of interface (solid/aqueous *vs* solid/air), incubation time, and chemical structure.

In Figure 8, we present a summary in terms of the depicted changes caused by various factors, such as the oxidation state of copper (Cu(I) *vs* Cu(II)), the type of interface (solid/aqueous *vs* solid/air), the incubation time, and the structure of the N-substituted analogues of 4-[(*N*H-R) (phosphono)-*S*-methyl]phenylboronic acid that functionalize the surface of the copper oxide NSs after adsorption. Briefly,

(1) 1-PBA interacts with the surface of CuONSs via only one aromatic ring—the phenyl ring, aligned at an angle to this surface, and lone pairs of electrons on the nitrogen and oxygen atoms of the −C(N)PO– fragment. At the Cu_2_ONP/water interface, the phosphonic acid group is moved away from the substrate surface and two aromatic rings of 1-PBA participate in the interaction with this substrate; the Ph ring adopts a vertical orientation with respect to the substrate surface, while the Ph_B(OH)_2__ ring is almost horizontal. The change of the interface from Cu_2_ONPs/water to CuO_2_NPs/air forces the molecule to “straighten up” so that the contact between the Ph_B(OH)_2__ ring and the substrate surface is maintained while the Ph ring moves away from this surface.
(2) By replacing the phenyl group with the benzyl group, the 2-PBA molecule interacts with the CuONS surface via the phosphonic acid group and the Ph ring, which moves away from the substrate surface and assumes a nearly vertical orientation with respect to that surface. 2-PBA adsorbs at the Cu_2_ONP/water and CuO_2_NP/air interfaces via the vertical Ph ring and the fragment −C(N)PO–, respectively.
(3) Further modification of the 1-PBA structure leads to another change in the bis{1-PBA} adsorption mode. That is, bis{1-PBA} is planar aligned near the CuONS surface, while the more or less vertical Ph_B(OH)_2__ rings are preserved. On the other hand, immediately after adsorption at the Cu_2_ONP/water interface, Ph_B(OH)_2__ is in contact with this interface and adopts a vertical orientation. In the following minutes after adsorption (up to 10 min), a reorientation is observed—the molecule lies down on the interface so that its two Ph_B(OH)_2__ rings adopt a more or less horizontal orientation with respect to the interface. Unlike at the Cu_2_ONP/air interface, the skeleton of bis{1-PBA} adopts an angular orientation.

## Abbreviations

Cu_2_ONPs: copper(I) oxide nanoparticles
CuONSs: copper(II) oxide nanostructures
1-PBA: 4-[(*N*-anilino)(phosphono)-*S*-methyl]phenylboronic acid
2-PBA: 4-[(*N*-benzylamino)(phosphono)-*S*-methyl]phenylboronic acid
bis{1-PBA}: bis{4-[(*N*-anilino)(phosphono)-*S*-methyl]phenylboronic acid}
SERS: surface-enhanced Raman spectroscopy

## References

[ref1] Estopiñá-Durán S.; Mclean E. B.; Donnelly L. J.; Hockin B. M.; Taylor J. E. Aryloboronic acid catalyzed C-alkylation and allylation reactions using benzylic alcohols. Org. Lett. 2020, 22, 7547–7551. 10.1021/acs.orglett.0c02736. 32959662 PMC8155392

[ref2] Irving A. M.; Vogels C. M.; Nikolcheva L. G.; Edwards J. P.; He X.-F.; Hamilton M. G.; Baerlocher M. O.; Baerlocher F. J.; Decken A.; Westcott S. A. Synthesis and antifungal and antibacterial bioactivity of cyclic diamines containing boronate esters. New J. Chem. 2003, 27, 1419–1424. 10.1039/b304500e.

[ref3] Ryu J. H.; Lee G. J.; Shih Y.-R. V.; Kim T.-I.; Varghese S. Phenylboronic Acid-polymers for Biomedical Applications. Curr. Med. Chem. 2019, 26, 6797–6816. 10.2174/0929867325666181008144436. 30295184

[ref4] Nemouchi S.; Boulcina R.; Carboni B.; Debache A. Phenylboronic acid as an efficient and convenient catalyst for a three-component synthesis of tetrahydrobenzo[b]pyrans. Compt. Rendus Chem. 2012, 15, 394–397. 10.1016/j.crci.2012.01.003.

[ref5] SeethaLekshmi N.; Pedireddi V. R. olid-State Structures of 4-Carboxyphenylboronic Acid and Its Hydrates. Cryst. Growth Des. 2007, 7, 944–949. 10.1021/cg060860c.

[ref6] Boron. Sensing, Synthesis and Supramolecular Self-Assembly; Meng L., Fossey J. S., James T. D., Eds.; Royal Society of Chemistry: Cambridge, 2015.

[ref7] Boronic Acids. Preparation and Applications in Organic Synthesis, Medicine and Materials, 2nd ed.; Hall D. G., Ed.; Wiley-VCH: Weinheim, 2011.

[ref8] Chu Y.; Wang D.; Wang K.; Liu Z.; Weston B.; Wang B. Fluorescent conjugate of sLe(x)-selective bisboronic acid for imaging application. Bioorg. Med. Chem. Lett. 2013, 23, 6307–6309. 10.1016/j.bmcl.2013.09.063. 24125887

[ref9] Geninatti Crich S.; Alberti D.; Szabo I.; Aime S.; Djanashvili K. MRI visualization of melanoma cells by targeting overexpressed sialic acid with a GdIII-dota-en-pba imaging reporter. Angew. Chem., Int. Ed. 2013, 52, 1161–1164. 10.1002/anie.201207131. 23225599

[ref10] Cambre J. N.; Sumerlin B. S. Biomedical applications of boronic acid polymers. Polymer 2011, 52, 4631–4643. 10.1016/j.polymer.2011.07.057.

[ref11] Lee J.; Kim J.; Lee Y. M.; Park D.; Im S.; Song E. H.; Park H.; Kim W. J. Self-assembled nanocomplex between polymerized phenylboronic acid and doxorubicin for efficient tumor-targeted chemotherapy. Acta Pharmacol. Sin. 2017, 38, 848–858. 10.1038/aps.2017.16. 28414203 PMC5520185

[ref12] Gao W.; Liang Y.; Peng X.; Hu Y.; Zhang L.; Wu H.; He B. In situ injection of phenylboronic acid based low molecular weight gels for efficient chemotherapy. Biomaterials 2016, 105, 1–11. 10.1016/j.biomaterials.2016.07.025. 27497056

[ref13] António J. P. M.; Russo R.; Carvalho C. P.; Cal P. M. S. D.; Gois P. M. P. Boronic acids as building blocks for the construction of therapeutically useful bioconjugates. Chem. Soc. Rev. 2019, 48, 3513–3536. 10.1039/c9cs00184k. 31157810

[ref14] James T. D.; Phillips M. D.; Shinkai S. Boronic Acids in Saccharide Recognition; RSC Publishing: Cambridge, 2006.

[ref15] Lacina K.; Skládal P.; James T. D. Boronic acids for sensing and other applications - a mini-review of papers published in 2013. Chem. Cent. J. 2014, 8, 60 10.1186/s13065-014-0060-5. 25371705 PMC4218984

[ref16] Li G.; Wen D. Sensing nanomaterials of wearable glucose sensors. Chin. Chem. Lett. 2021, 32, 221–228. 10.1016/j.cclet.2020.10.028.

[ref17] Taguchi M.; Ptitsyn A.; McLamore E. S.; Claussen J. C. Nanomaterial-mediated Biosensors for Monitoring Glucose. J. Diabetes Sci. Technol. 2014, 8, 403–411. 10.1177/1932296814522799. 24876594 PMC4455391

[ref18] Brooks W. L. A.; Sumerlin B. S. Synthesis and Applications of Boronic Acid-Containing Polymers: From Materials to Medicine. Chem. Rev. 2016, 116, 1375–1397. 10.1021/acs.chemrev.5b00300. 26367140

[ref19] Wulff G.; Lauer M.; Böhnke H. Rapid Proton Transfer as Cause of an Unusually Large Neighboring Group Effect. Angew. Chem., Int. Ed. Engl. 1984, 23, 741–742. 10.1002/anie.198407411.

[ref20] Yang X.; Lee M.-C.; Sartain F.; Pan X.; Lowe C. R. Designed Boronate Ligands for Glucose-Selective Holographic Sensors. Chem. - Eur. J. 2006, 12, 8491–8497. 10.1002/chem.200600442. 16906615

[ref21] Hughes M. P.; Smith B. D. Enhanced Carboxylate Binding Using Urea and Amide-Based Receptors with Internal Lewis Acid Coordination: a Cooperative Polarization Effect. J. Org. Chem. 1997, 62, 4492–4499. 10.1021/jo9702249. 11671780

[ref22] Bhat K. L.; Howard N. J.; Rostami H.; Lai J. H.; Bock C. W. Intramolecular Dative Bonds Involving Boron with Oxygen and Nitrogen in Boronic Acids and Esters: a Computational Study. J. Mol. Struct. 2005, 723, 147–157. 10.1016/j.theochem.2005.01.033.

[ref23] Beers S. A.; Schwender C. F.; Loughney D. A.; Malloy E.; Demarest K.; Jordan J. Phosphatase inhibitors-III. Benzylaminophosphonic acids as potent inhibitors of human prostatic acid phosphatase. Bioorg. Med. Chem. 1996, 4, 1693–1701. 10.1016/0968-0896(96)00186-1. 8931939

[ref24] Pawełczak M.; Nowak K.; Kafarski P. Synthesis of phosphonodipeptides, inhibitors of cathepsin C. Phosphourus Sulfur 1998, 132, 65–71.

[ref25] Vovk A. I.; Mischenko I. M.; Tanchuk V. Y.; Kachkovskii G. A.; Sheiko S. Y.; Kolodyazhnyi O. I.; Kukhar V. P. Stereoselectivity of binding of α-(N-benzylamino)benzylphosphonic acids to prostatic acid phosphatase. Bioorg. Med. Chem. Lett. 2008, 18, 4620–4623. 10.1016/j.bmcl.2008.07.021. 18672366

[ref26] Cao K.; Jiang X.; Yan S.; Zhang L.; Wu W. Phenylboronic acid modified silver nanoparticles for colorimetric dynamic analysis of glucose. Biosens. Bioelectron. 2014, 52, 188–195. 10.1016/j.bios.2013.08.046. 24055932

[ref27] Pham X.-H.; Shim S.; Kim T.-H.; Hahm E.; Kim H.-M.; Rho W.-Y.; Jeong D. H.; Lee Y.-S.; Jun B.-H. Glucose detection using 4-mercaptophenyl boronic acid-incorporated silver nanoparticles-embedded silica-coated graphene oxide as a SERS substrate. Biochip J. 2017, 11, 46–56. 10.1007/s13206-016-1107-6.

[ref28] Wu W.; Chen S.; Hu Y.; Zhou S. A fluorescent responsive hybrid nanogel for closed-loop control of glucose. J. Diabetes Sci. Technol. 2012, 6, 892–901. 10.1177/193229681200600421. 22920816 PMC3440161

[ref29] Deng R.; Yue J.; Qu H.; Liang L.; Sun D.; Zhang J.; Liang C.; Xu W.; Xu S. Glucose-bridged silver nanoparticle assemblies for highly sensitive molecular recognition of sialic acid on cancer cells via surface-enhanced raman scattering spectroscopy. Talanta 2018, 179, 200–206. 10.1016/j.talanta.2017.11.006. 29310222

[ref30] Liu A.; Peng S.; Soo J. C.; Kuang M.; Chen P.; Duan H. Quantum dots with phenylboronic acid tags for specific labeling of sialic acids on living cells. Anal. Chem. 2011, 83, 1124–1130. 10.1021/ac1028853. 21182248

[ref31] Zhang X.; Chen B.; He M.; Zhang Y.; Peng L.; Hu B. Boronic acid recognition based-gold nanoparticle-labeling strategy for the assay of sialic acid expression on cancer cell surface by inductively coupled plasma mass spectrometry. Analyst 2016, 141, 1286–1293. 10.1039/c5an02402a. 26811850

[ref32] Wu W.; Zhou T.; Shen J.; Zhou S. Optical detection of glucose by CdS quantum dots immobilized in smart microgels. Chem. Commun. 2009, 4390–4392. 10.1039/b907348e. 19597602

[ref33] Yetisen A. K.; Montelongo Y.; da Cruz Vasconcellos F.; Martinez-Hurtado J. L.; Neupane S.; Butt H.; Qasim M. M.; Blyth J.; Burling K.; Carmody J. B.; Evans M.; Wilkinson T. D.; Kubota L. T.; Monteiro M. J.; Lowe C. R. Reusable, robust, and accurate laser-generated photonic nanosensor. Nano Lett. 2014, 14, 3587–3593. 10.1021/nl5012504. 24844116

[ref34] Karlsson A. L.; Toprak M. S.; Fadeel B. Toxicity of metal and metal oxide nanoparticles. Handbook on the Toxicology of Metals 4E; Elsevier, 2015.

[ref35] Horie M.; Fujita K. Toxicity of metal oxides nanoparticles. Adv. Mol. Toxicol. 2011, 5, 145–178. 10.1016/b978-0-444-53864-2.00004-9.

[ref36] Benguigui M.; Weitz I. S.; Timaner M.; Kan T.; Shechter D.; Perlman O.; Sivan S.; Raviv Z.; Azhari H.; Shaked Y. Copper oxide nanoparticles inhibit pancreatic tumor growth primarily by targeting tumor initiating cells. Sci. Rep. 2019, 9, 12613 10.1038/s41598-019-48959-8. 31471546 PMC6717199

[ref37] Gnanavel V.; Palanichamy V.; Roopan S. M. Biosynthesis and characterization of coper oxide nanoparticles and its anticancer activity on human colon cancer cell lines (HCT-116). J. Photochem. Photobiol., B 2017, 171, 133–138. 10.1016/j.jphotobiol.2017.05.001. 28501691

[ref38] Nagajyothi P. C.; Muthuraman P.; Sreekanth T. V. M.; Kim D. H.; Shim J. Green synthesis: In-vitro anticancer activity of copper oxide nanoparticles against human cervical carcinoma cells. Arab. J. Chem. 2017, 10, 215–225. 10.1016/j.arabjc.2016.01.011.

[ref39] Kayran Y. U.; Jambrec D.; Schuhmann W. Nanostructured DNA microarrays for dual SERS and electrochemical read-out. Electroanalysis 2018, 31, 267–272. 10.1002/elan.201800579.

[ref40] Ambartsumyan O.; Gribanyov D.; Kukushkin V.; Kopylov A.; Zavyalova E. SERS-based biosensors for virus determination with oligonucleotides as recognition elements. Int. J. Mol. Sci. 2020, 21, 3373 10.3390/ijms21093373. PMC7247000 32397680

[ref41] Potara M.; Boca S.; Licarete E.; Damert A.; Alupei M.-C.; Chiriac M. T.; Popescu O.; Schmidt U.; Astilean S. Chitosan-coated triangular silver nanoparticles as a novel class of biocompatible, highly sensitive plasmonic platforms for intracellular SERS sensing and imaging. Nanoscale 2013, 5, 6013–6022. 10.1039/c3nr00005b. 23715524

[ref42] Fikiet M. A.; Khandasammy S. R.; Mistek E.; Ahmed Y.; Halámková L.; Bueno J.; Lednev I. K. Surface-enhanced Raman spectroscopy: A review of recent applications in forensic science. Spectrochim. Acta, Part A 2018, 197, 255–260. 10.1016/j.saa.2018.02.046. 29496406

[ref43] Guerrini L.; Pazos-Perez N.; Garcia-Rico E.; Alvarez-Puebla R. Cancer characterization and diagnosis with SERS-encoded particles. Cancer Nanotechnol. 2017, 8, 5 10.1186/s12645-017-0031-3.

[ref44] Hering K.; Cialla D.; Ackermann K.; Dörfer T.; Möller R.; Schneidewind H.; Mattheis R.; Fritzsche W.; Rösch P.; Popp J. SERS: a versatile tool in chemical and biochemical diagnostic. Anal. Bioanal. Chem. 2008, 390, 113–124. 10.1007/s00216-007-1667-3. 18000657

[ref45] Limo M. J.; Sola-Rabada A.; Boix E.; Thota V.; Westcott Z. C.; Puddu V.; Perry C. C. Interactions between metal oxides and biomolecules: from fundamental understanding to applications. Chem. Rev. 2018, 118, 11118–11193. 10.1021/acs.chemrev.7b00660. 30362737

[ref46] Pienpinijtham P.; Proniewicz E.; Kim Y.; Ozaki Y.; Lombardi J. R.; Proniewicz L. M. Molecular orientation of neurotensin and its single-site mutants on colloidal silver surface: SERS Studies. J. Phys. Chem. C 2012, 116, 16561–16572. 10.1021/jp304205d.

[ref47] Zheng Y.; Rosa L.; Thai T.; Ng S. H.; Juodkazis S.; Bach U. Phase controlled SERS enhancement. Sci. Rep. 2017, 9, 744 10.1038/s41598-018-36491-0. PMC6346009 30679465

[ref48] Markin A. V.; Markina N. E.; Popp J.; Cialla-May D. Copper nanostructures for chemical analysis using surface-enhance Raman spectroscopy. Trends Anal. Chem. 2018, 108, 247–259. 10.1016/j.trac.2018.09.004.

[ref49] Han X. X.; Ji W.; Zhao B.; Ozaki Y. Semiconductor-enhanced Rama scattering: active nanomaterials and applications. Nanoscale 2017, 9, 4847–4861. 10.1039/c6nr08693d. 28150834

[ref50] Młynarz P.; Rydzewska A.; Pokładek Z. Preparation of a Novel Group of Hybrid Compounds N-Benzyl Aminoboronbenzylphosphonic and N,N’-Ethylenedi(aminoboronbenzyl phosphonic) Acids. J. Organomet. Chem. 2011, 696, 457–460. 10.1016/j.jorganchem.2010.09.002.

[ref51] Starowicz M. Electrochemical synthesis of copper oxide particles with controlled oxidation state, shape and size. Mater. Res. Express 2019, 6, 0850a3 10.1088/2053-1591/ab239d.

[ref52] Stypuła B.; Banaś J.; Starowicz M.; Krawiec H.; Bernasik A.; Janas A. Production of nanoparticles of copper compounds by anodic dissolution of copper in organic solvents. J. Appl. Electrochem. 2006, 36, 1407–1414. 10.1007/s10800-006-9233-9.

[ref53] Borgohain K.; Murase N.; Mahamuni S. Synthesis and properties of Cu2O quantum particles. J. Appl. Phys. 2002, 92, 1292–1297. 10.1063/1.1491020.

[ref54] Pestryakov A. N.; Petranovskii V. P.; Kryazhov A.; Ozhereliev O.; Pfänder N.; Knop-Gericke A. Study of copper nanoparticles formation on supports of different nature by UV-Vis diffuse reflectance spectroscopy. Chem. Phys. Lett. 2004, 385, 173–176. 10.1016/j.cplett.2003.12.077.

[ref55] Yin M.; Wu C.-K.; Lou Y.; Burda C.; Koberstein J. T.; Zhu Y.; O’Brien S. Copper Oxide Nanocrystals. J. Am. Chem. Soc. 2005, 127, 9506–9511. 10.1021/ja050006u. 15984877

[ref56] Cakir D. Enhanced Raman signatures on copper based-materials. Thesis, HAL archives-ouvertes, fr Université Montpellier, 2017. https://tel.archives-ouvertes.fr/tel-01944233.

[ref57] Zimbovskiy D. S.; Gavrilov A. I.; Churagulov B. R. Synthesis of copper oxides films via anodic oxidation of copper foil followed by thermal reduction. Mater. Sci. Eng. 2018, 347, 012010 10.1088/1757-899x/347/1/012010.

[ref58] Anu A.; Khadara M. A. Grain size tuning of nanostructured Cu_2_O films through vapour phase supersaturation control and their characterization for practical applications. AIP Adv. 2015, 5, 097176 10.1063/1.4932087.

[ref59] Bello A.; Dodoo-Arhin D.; Makgopa K.; Fabiane M.; Manyala N. Surfactant Assisted Synthesis of Copper Oxide (CuO) Leaf-like Nanostructures for Electrochemical Applications. Am. J. Mat. Sci. 2014, 4, 64–73. 10.5923/j.materials.20140402.03.

[ref60] Zhao B.; Liu P.; Zhuang H.; Jiao Z.; Fang T.; Xu W.; Lu B.; Jiang Y. Hierarchical self-assembly of microscale leaf-like CuO on graphene sheets for high-performance electrochemical capacitors. J. Mater. Chem. A 2013, 1, 367–373. 10.1039/c2ta00084a.

[ref61] Zhang K.; Rossi C.; Tenaillea Ch.; Alphonse P.; Chane-Ching J.-Y. Synthesis of large-area and aligned copper oxide nanowires from copper thin film on silicon substrat. Nanotechnology 2007, 18, 275607 10.1088/0957-4484/18/27/275607.

[ref62] Kozak D. S.; Sergiienko R. A.; Shibata E.; Iizuka A.; Nakamura T. Non-electrolytic synthesis of copper oxide/carbon nanocomposite by surface plasma in super-dehydrated ethanol. Sci. Rep. 2016, 6, 21178 10.1038/srep21178. 26880365 PMC4754732

[ref63] Dhineshbabu N. R.; Rajendran V.; Nithyavathy N.; Vetumperumal R. Study of structural and optical properties of cupric oxide nanoparticles. Appl. Nanosci. 2016, 6, 933–939. 10.1007/s13204-015-0499-2.

[ref64] Tahir D.; Tougaard S. Electronic and optical properties of Cu, CuO and Cu2O studied by electron spectroscopy. J. Phys.: Condens. Matter 2012, 24, 175002–175010. 10.1088/0953-8984/24/17/175002. 22475683

[ref65] Morales J.; Espinos J. P.; Caballero A.; Gonzalez-Elipe A. R.; Mejias J. A. XPS Study of Interface and Ligand Effects in Supported Cu2O and CuO Nanometric Particles. J. Phys. Chem. B 2005, 109, 7758–7765. 10.1021/jp0453055. 16851901

[ref66] Chan G. H.; Zhao J.; Hicks E. M.; Schatz G. C.; Van Duyne R. P. Plasmonic Properties of Copper Nanoparticles Fabricated by Nanosphere Lithography. Nano Lett. 2007, 7, 1947–1952. 10.1021/nl070648a. 16178265

[ref67] Ragavan K. V.; Rastogi N. K. Graphene–copper oxide nanocomposite with intrinsic peroxidase activity for enhancement of chemiluminescence signals and its application for detection of Bisphenol-A. Sens. Actuators, B 2016, 229, 570–580. 10.1016/j.snb.2016.02.017.

[ref68] El-Trass A.; ElShamy H.; El-Mehasseb I.; El-Kemary M. CuO nanoparticles: Synthesis, characterization, optical properties and interaction with amino acids. Appl. Surf. Sci. 2012, 258, 2997–3001. 10.1016/j.apsusc.2011.11.025.

[ref69] Hagemann H.; Bill H.; Sadowski W.; Walker E.; François M. Raman spectra of single crystal CuO. Solid State Commun. 1990, 73, 447–451. 10.1016/0038-1098(90)90048-g.

[ref70] Tran T. H.; Nguyen V. T. Phase transition of Cu2O to CuO nanocrystals by selective laser heating. Mat. Sci. Semicon. Proc. 2016, 46, 6–9. 10.1016/j.mssp.2016.01.021

[ref71] Zheng S.-F.; Hu J.-S.; Zhong L.-S.; Song W.-G.; Wan L.-J.; Guo Y.-G. Introducing Dual Functional CNT Networks into CuO Nanomicrospheres toward Superior Electrode Materials for Lithium-Ion Batteries. Chem. Mater. 2008, 20, 3617–3622. 10.1021/cm7033855.

[ref72] Piergies N.; Proniewicz E.; Kudelski A.; Rydzewska A.; Kim Y.; Andrzejak M.; Proniewicz L. M. Fourier Transform Infrared and Raman and Surface-Enhanced Raman Spectroscopy Studies of a Novel Group of Boron Analogues of Aminophosphonic Acids. J. Phys. Chem. A 2012, 116, 10004–10014. 10.1021/jp307064p. 22988982

[ref73] Podstawka E.; Borszowska R.; Grabowska M.; Drąg M.; Kafarski P.; Proniewicz L. M. Investigation of Molecular Structure and Adsorption Mechanism of Phosphonodipeptides by infrared, Raman, and surface enhanced Raman spectroscopy. Surf. Sci. 2005, 599, 207–220. 10.1016/j.susc.2005.09.048.

[ref74] Li S.; Zhou Q.; Chu W.; Zhao W.; Zheng J. Surface-enhanced Raman Scattering Behaviours of 4-Mercaptophenyl boronic Acid on Assembled Silver Nanoparticles. Phys. Chem. Chem. Phys. 2015, 17, 17638–17645. 10.1039/c5cp02409a. 26080999

[ref75] Marasovic M.; Ivankovic S.; Stojkovic R.; Djermic D.; Galic B.; Milos M. In vitro and in vivo antitumour effects of phenylboronic acid against mouse mammary adenocarcinoma 4T1 and squamous carcinoma SCCVII cells. J. Enzyme Inhib. Med. Chem. 2017, 32, 1299–1304. 10.1080/14756366.2017.1384823. 29072095 PMC6010135

[ref76] Bradke T. M.; Hall C.; Carper S. W.; Plopper G. E. Phenylboronic acid selectively inhibits human prostate and breast cancer cell migration and decreases viability. Cell Adh. Migr. 2008, 2, 153–160. 10.4161/cam.2.3.6484. 19262119 PMC2634091

[ref77] Le Ru E. C.; Blackie E.; Meyer M.; Etchegoin P. G. Surface Enhanced Raman Scattering Enhancement Factors: A Comprehensive Study. J. Phys. Chem. C 2007, 111, 13794 10.1021/jp0687908.

[ref78] Proniewicz E.; Tąta A.; Wójcik A.; Starowicz M.; Pacek J.; Molenda M. SERS activity and spectroscopic properties of Zn and ZnO nanostructures obtained by electrochemical and green chemistry methods for applications in biology and medicine. Phys. Chem. Chem. Phys. 2020, 22, 28100–28114. 10.1039/d0cp03517c. 33289732

[ref79] Proniewicz E.; Tąta A.; Szkudlarek A.; Świder J.; Molenda M.; Starowicz M.; Ozaki Y. Electrochemically synthetized γ-Fe_2_O_3_ nanoparticles as peptide carriers and sensitive and reproducible SERS biosensors. Comparison of adsorption on Fe_2_O_3_ versus Fe. Appl. Surf. Sci. 2019, 495, 143578 10.1016/j.apsusc.2019.143578.

[ref80] Moskovits M.; Suh J. S. Surface geometry change in 2-naphthoic acid adsorbed on silver. J. Phys. Chem. 1988, 92, 6327 10.1021/j100333a030.

[ref81] Greaves S. J.; Griffith W. P. Vibrational spectra of catechol, catechol-d2 and -d6 and the catecholate monoanion. Spectrochim. Acta, Part A 1991, 47, 133–140. 10.1016/0584-8539(91)80185-l.

[ref82] Arenas J. F.; Woolley M. S.; Tocón I. L.; Otero J. C.; Marcos J. I. Complete analysis of the surface-enhanced Raman scattering of pyrazine on the silver electrode on the basis of a resonant charge transfer mechanism involving three states. J. Chem. Phys 2000, 112, 7669–7683. 10.1063/1.481361.

[ref83] Arenas J. F.; Soto J.; Tocón I. L.; Fernández D. J.; Otero J. C.; Marcos J. I. The role of charge-transfer states of the metal-adsorbate complex in surface-enhanced Raman scattering. J. Chem. Phys 2002, 116, 7207–7216. 10.1063/1.1450542.

[ref84] Castro J. L.; López Ramirez M. R.; López Tocón I.; Otero J. C. Vibrational study of the metal-adsorbate interaction of phenylacetic acid and alpha-phenylglycine on silver surfaces. J. Colloid Interface Sci. 2003, 263, 357–363. 10.1016/s0021-9797(03)00257-1. 12909024

[ref85] Kolben T. M.; Kraft F.; Kolben T.; Goess C.; Semmlinger A.; Dannecker C.; Schmoeckel E.; Mayr D.; Sommer N. N.; Mahner S.; Jeschke U. Expression of Sialyl Lewis a, Sialyl Lewis x, Lewis y, Gal-3, Gal-7, STMN1 and p16 in cervical dysplasia. Future Oncol. 2017, 13, 145–157. 10.2217/fon-2016-0259. 27646625

[ref86] Springsteen G.; Wang B. Alizarin Red S. as a general optical reporter for studying the binding of boronic acids with carbohydrates. Chem. Commun. 2001, 17, 1608–1609. 10.1039/b104895n. 12240405

[ref87] Tharmaraj V.; Pitchumani K. D-Glucorse sensing by (E)-(4-((pyren-1-ylmethylene)amino)phenyl)boronic acid via a photoinduced electron transfer (PET) mechanism. RSC Adv. 2013, 3, 11566–11570. 10.1039/c3ra40544c.

[ref88] Neupane L. N.; Lohani C. R.; Kim J.; Lee K.-H. A dual role of phenylboronic acid as a receptor for carbohydrates as well as a quencher for neighboring pyrene fluorophore. Tetrahedron 2013, 69, 11057–11063. 10.1016/j.tet.2013.11.023.

[ref89] Wang Z.; Lei H.; Feng L. A facile channel for D-glucose detection in aqueous solution. Spectrochim. Acta, Part A 2013, 114, 293–297. 10.1016/j.saa.2013.05.089. 23778168

[ref90] Huang Y.-J.; Ouyang W.-J.; Wu X.; Li Z.; Fossey J. S.; James T. D.; Jiang Y.-B. Glucose sensing via aggregation and the use of “Knock-Out” binding to improve selectivity. J. Am. Chem. Soc. 2013, 135, 1700–1703. 10.1021/ja311442x. 23317305

[ref91] You T.; Niwa O.; Tomita M.; Ando H.; Suzuki M.; Hirono S. Characterization and electrochemical properties of highly dispersed copper oxide/hydroxide nanoparticles in graphite-like carbon films prepared by RF sputtering method. Electrochem. Commun. 2002, 4, 468–471. 10.1016/s1388-2481(02)00340-5.

[ref92] Farrell S. T.; Breslin C. B. Oxidation and photo-induced oxidation of glucose at a polyaniline film modified by copper particles.Electrochim. Acta 2004, 49, 4497–4503. 10.1016/j.electacta.2004.05.007

[ref93] Xu Q.; Zhao Y.; Xu J. Z.; Zhu J.-J. Preparation of functionalized copper nanoparticles and fabrication of a glucose sensor. Sens. Actuators, B 2006, 114, 379–386. 10.1016/j.snb.2005.06.005.

[ref94] Maaoui H.; Teodoresu F.; Wang Q.; Pan G.-H.; Addad A.; Chtourou R.; Szunerits S.; Boukherroub R. Non-Enzymatic Glucose Sensing Using Carbon Quantum Dots Decorated with Copper Oxide Nanoparticles. Sensors 2016, 16, 1720 10.3390/s16101720. PMC5087507 27763533

[ref95] Wang W.; Zhang L.; Tong S.; Li X.; Song W. Three-dimensional network films of electrospun copper oxide nanofibers for glucose determination. Biosens. Bioelectron. 2009, 25, 708–714. 10.1016/j.bios.2009.08.013. 19733046

[ref96] Xia L.; Xu L.; Song J.; Xu R.; Liu D.; Dong B.; Song H. CdS quantum dots modified CuO inverse opal electrodes for ultrasensitive electrochemical and photoelectrochemical biosensor. Sci. Rep. 2015, 5, 10838 10.1038/srep10838. 26042520 PMC4455289

[ref97] Luque G.; Rodriguez M.; Rivas G. Glucose biosensors based on the immobilization of copper oxide and glucose oxidase within a carbon paste matrix. Talanta 2005, 66, 467–471. 10.1016/j.talanta.2004.07.019. 18970008

[ref98] Halbus A. F.; Horozov T. S.; Paunov V. N. Strongly Enhanced Antibacterial Action of Copper Oxide Nanoparticles with Boronic Acid Surface Functionality. ACS Appl. Mater. Interfaces 2019, 11, 12232–12243. 10.1021/acsami.8b21862. 30892875

[ref99] Henry P.; Halbus A. F.; Athab Z. H.; Paunov V. N. Enhanced Antimould Action of Surface Modified Copper Oxide Nanoparticles with Phenylboronic Acid Surface Functionality. Biomimetics 2021, 6, 19 10.3390/biomimetics6010019. 33804236 PMC8006150

[ref100] Paolucci C.; Khurana I.; Parekh A. A.; Li S.; Shih A. J.; Li H.; Di Iorio J. R.; Albarracin-Caballero J. D.; Yezerets A.; Miller J. T.; Delgass W. N.; Ribeiro F. H.; Schneider W. F.; Gounder R. Dynamic multinuclear sites formed by mobilized copper ions in NO_x_ selective catalytic reduction. Science 2017, 357, 898–903. 10.1126/science.aan5630. 28818971

[ref101] Li M.; Ge H.; Arrowsmith R. L.; Mirabello V.; Botchway S. W.; Zhu W.; Pascu S. I.; James T. D. Ditopic boronic acid and imine-based naphthalimide fluorescence sensor for copper(ii). Chem. Commun. 2014, 50, 11806–11809. 10.1039/c4cc03453h. 24919009

[ref102] Maity D.; Hari N.; Mohanta S. A Bis(Boronic Ester)-Based Fluorogenic and Chromogenic Sensor for F^–^ and Cu^2+^. ChemistrySelect 2017, 2, 9037–9045. 10.1002/slct.201700891.

